# Different Modes of Action of Genetic and Chemical Downregulation of Histone Deacetylases with Respect to Plant Development and Histone Modifications

**DOI:** 10.3390/ijms20205093

**Published:** 2019-10-14

**Authors:** Gabriela Lochmanová, Ivana Ihnatová, Hana Kuchaříková, Sylva Brabencová, Dagmar Zachová, Jiří Fajkus, Zbyněk Zdráhal, Miloslava Fojtová

**Affiliations:** 1 Mendel Centre for Plant Genomics and Proteomics, Central European Institute of Technology, Masaryk University, 62500 Brno, Czech Republic; gabriela.lochmanova@ceitec.muni.cz (G.L.); hana.kucharikova@ceitec.muni.cz (H.K.); 258751@mail.muni.cz (S.B.); dagmar.zachova@ceitec.muni.cz (D.Z.); fajkus@sci.muni.cz (J.F.); 2 RECETOX, Faculty of Science, Masaryk University, 62500 Brno, Czech Republic; ivana.ihnatova@recetox.muni.cz; 3 Laboratory of Functional Genomics and Proteomics, National Centre for Biomolecular Research, Faculty of Science, Masaryk University, 62500 Brno, Czech Republic

**Keywords:** *Arabidopsis thaliana*, epigenetics, histone, mass spectrometry, post-translational modifications, sodium butyrate, trichostatin A

## Abstract

A high degree of developmental plasticity enables plants to adapt to continuous, often unfavorable and unpredictable changes in their environment. At the molecular level, adaptive advantages for plants are primarily provided by epigenetic machinery including DNA methylation, histone modifications, and the activity of noncoding RNA molecules. Using a mass spectrometry-based proteomic approach, we examined the levels of acetylated histone peptide forms in Arabidopsis plants with a loss of function of histone deacetylase 6 (HDA6), and in plants germinated in the presence of HDA inhibitors trichostatin A (TSA) and sodium butyrate (NaB). Our analyses revealed particular lysine sites at histone sequences targeted by the HDA6 enzyme, and by TSA- and NaB-sensitive HDAs. Compared with plants exposed to drugs, more dramatic changes in the overall profiles of histone post-translational modifications were identified in *hda6* mutants. However, loss of HDA6 was not sufficient by itself to induce hyperacetylation to the maximum degree, implying complementary activities of other HDAs. In contrast to *hda6* mutants that did not exhibit any obvious phenotypic defects, the phenotypes of seedlings exposed to HDA inhibitors were markedly affected, showing that the effect of these drugs on early plant development is not limited to the modulation of histone acetylation levels.

## 1. Introduction

The dynamics of chromatin structure, reflecting changes related to specific developmental stages and various environmental conditions, is crucial for the maintenance of essential functions of each cell in a multicellular organism. Nucleosomes, the basic chromatin units, consisting of DNA wrapped around histone octamers, are sophisticatedly arranged in a higher-order supramolecular structure that is expected to allow for all processes in which DNA is involved, such as DNA replication, transcription and repair. Reversible modifications of protruding histone N-termini are important for modulating the strength of association between histones and DNA, and for recruitment of chromatin-associated proteins. Modification of positively charged amino acids lysine (K) and arginine (R), which are frequently located at the N-termini of H3 and H4 histones, by addition of acetyl group results in neutralization of their positive charge, thus weakening electrostatic bonds between histones and phosphates in DNA and resulting in a more relaxed chromatin structure. Acetylated regions of chromatin are thus generally associated with transcriptionally active euchromatin [1,2], while histone hypoacetylation leads to the formation of more condensed heterochromatin [2,3,4]. Enzymes catalyzing loading on and removal of acetyl group from histones, histone acetyltransferases (HATs) and histone deacetylases (HDAs), respectively, play important roles in crucial processes in plant development, e.g., embryogenesis, timing of flowering and senescence, stress response and adaptation [5,6,7,8].

Two basic approaches are commonly utilized for functional studies of histone acetylation in the model plant *Arabidopsis thaliana*; analysis of mutants with a T-DNA insertion in genes encoding HATs or HDAs, or treatment of plants with chemical compounds that can modulate the activities of these enzymes. Regarding *HDAs*, 18 paralogs grouped into three types were identified; 12 RPD3 (reduced potassium deficiency 3)-like genes, 2 SIR2 (silent information regulator 2) genes, and four plant-specific genes so-called HD-tuins [9,10]. *HDA6* is a member of the RPD3-like superfamily. Two *HDA6* mutant alleles were described and analyzed; *hda6-6* obtained by EMS mutagenesis [11], and *hda6-7* with the T-DNA insertion [12]. Involvement of HDA6 in the maintenance of transcriptional gene silencing and nucleolar dominance [13,14,15], in the response to stress conditions [16,17], and in flowering-related processes [18] was described. As *hda6* mutants exhibited no crucial developmental abnormalities, other HDA members are assumed to act redundantly and compensate for HDA6 loss of function. Correspondingly, in *hda6/hda19* double mutants (with HDA19 being another prominent member of the RPD-3 superfamily), strong growth arrest during germination and formation of embryo-like structures were reported [6], and this phenotype was more severe compared to *hda19* single mutants [7]. In this respect, studies of plants exposed to HDA inhibitors, drugs that are able to influence the activity of several HDAs due to common structural domains of these enzymes, seem promising. Trichostatin A (TSA) and sodium butyrate (NaB) are the most commonly used inhibitors of RPD3-like HDAs. Using crystallographic studies, it was shown that TSA was able to insert its aliphatic chain into the hydrophobic cleft located in the catalytically active domain of HDAs [19]. A similar mode of action was proposed for NaB, because two NaB molecules could occupy the hydrophobic cleft in a similar way as demonstrated for TSA. Nevertheless, NaB is a non-competitive inhibitor of HDAs, thus does not associate with the substrate-binding region [20]. Details of NaB inhibition activity remain still enigmatic. As consequences of TSA exposure, de-repression of silenced rRNA genes [3], growth arrest and elevated transcription of embryogenesis-related genes [6] were reported in *A. thaliana*, and growth of primary roots was inhibited in poplar [21]. Interestingly, although inhibition of HDAs and increased levels of histone acetylation are supposed to lead to transcriptional up-regulation, significantly more genes were down-regulated than up-regulated in the latter study, suggesting that acetylation of histones may influence gene expression either positively or negatively [21]. Similarly to TSA, NaB activated silenced rRNA genes [3,22], and induced defects in seed germination and seedling growth were correlated with elevated transcription of base-excision repair genes in alfalfa [23].

For analyses of histone modifications, many methods are used, with mass spectrometry (MS) being widely recognized in the last years as an important tool for these studies. MS enables both qualitative and quantitative analysis of the distribution of histone post-translational modifications (PTMs). Although standard MS approaches are generally applicable for analysis of histones isolated from both mammalian and plant tissues, extraction of plant histones is complicated due to the presence of many secondary metabolites and other compounds contaminating histone extracts. Problems associated with histone solubility are also common. These difficulties are reflected in markedly more limited utilization of MS for analyses of PTMs of plant histones compared to mammals. To overcome these obstacles, we presented a straightforward approach for preparation of plant histone samples for MS analysis based on filter-aided sample preparation coupled with histone propionylation [24].

Here, we monitored phenotype and compared the level of acetylated and non-acetylated histone peptide forms in *A. thaliana* plants with loss of function of the gene encoding HDA6, and plants germinated in the presence of HDA inhibitors TSA or NaB. Our results suggest that the strong and dose-dependent effects of TSA and NaB on early plant development are complex and are not restricted to the ability of these drugs to influence the levels of histone acetylation via inhibition of activity of RPD3-like HDA enzymes.

## 2. Results

### 2.1. Strong Phenotype of Seedlings Germinated in the Presence of HDA Inhibitors Is Recovered during Cultivation of Plants in the Soil

**Figure 1:**
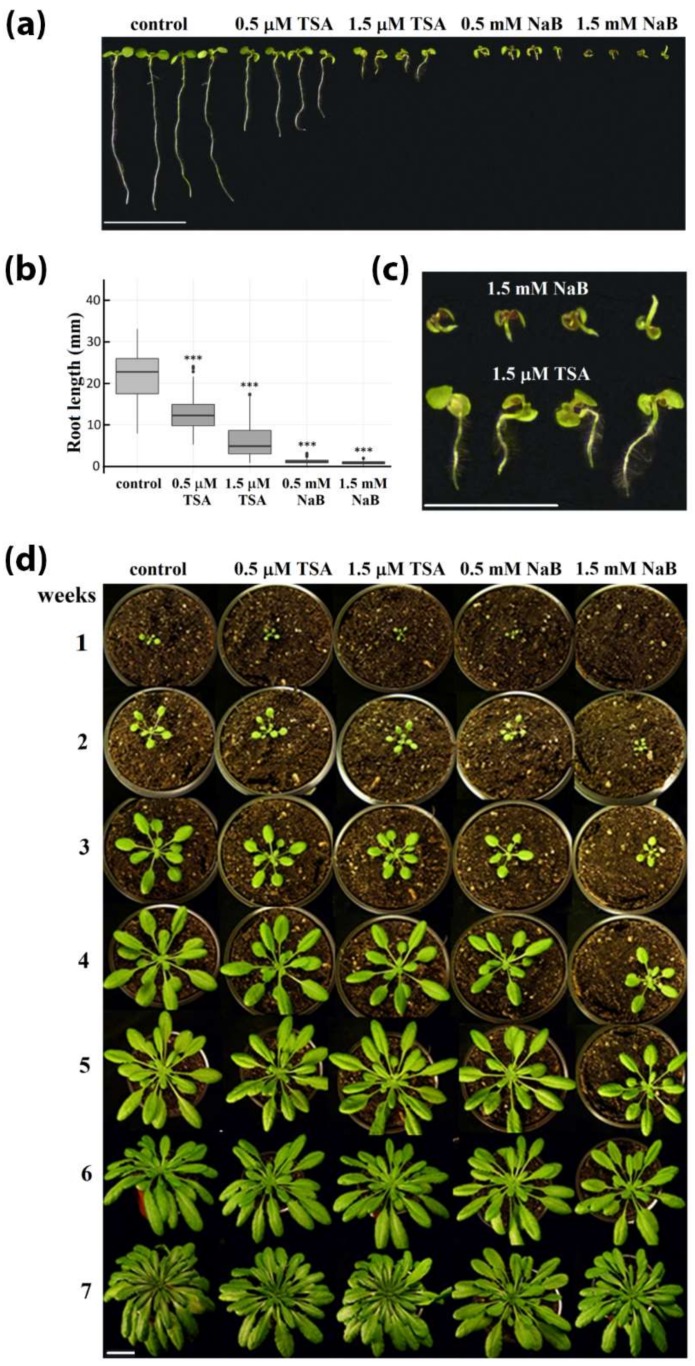
Effect of histone deacetylase (HDA) inhibitors on Arabidopsis growth and development. (**a**) Morphology of 7-day-old seedlings germinated in the presence of different concentrations of HDA inhibitors. Bar = 1 cm. (**b**) Lengths of primary roots in control and HDA inhibitor-treated seedlings. The box-plots show extremes, interquartile ranges and medians (*n* = 30). Statistically significant differences corresponding to *p* < 0.001 are marked by asterisks. (**c**) Detail of morphology of 7-day-old seedlings germinated in the presence of 1.5 mM NaB or 1.5 μM TSA. Bar = 0.5 cm. (**d**) Phenotype recovery of plants germinated in the presence of HDA inhibitors during the cultivation in the soil. Bar = 2 cm.

Morphological changes of *A. thaliana* plants associated with genetic (plants with loss of function of HDA6) or chemical (germination in the presence of HDA inhibitors, NaB or TSA) modulation of histone acetylation levels were examined. No clearly consistent alterations of phenotype were observed compared with wild-type Col-0 plants in both *hda6* lines germinated for 7 days followed by 7 weeks of cultivation in the soil, except for a slightly delayed flowering, in accordance with previously published observations [25]. However, when *A. thaliana* Col-0 seedlings were cultivated on MS medium supplemented with HDA inhibitors, a dose-dependent inhibition of early seedling development was observed (Figure 1a). One of the most distinctive symptoms was inhibition of root elongation, which was significantly more pronounced in seedlings germinated in the presence of NaB. The lengths of primary roots of 30 representatives per line were measured using the ImageJ software. The differences in their lengths compared to controls were significant in all HDA inhibitor-treated seedlings (*p* < 0.001; Figure 1b). NaB-exposed seedlings exhibited signs of agravitropism and chlorosis, and TSA exposure increased root hair length and density (Figure 1c). In spite of substantial developmental defects in seedlings germinated in the presence of HDA inhibitors, following 7 weeks of cultivation in soil without inhibitors, plants were morphologically indistinguishable from untreated controls, indicating recovery of plant phenotype (Figure 1d).

### 2.2. Distinct Alterations in Histone PTMs Are Hidden under a Mild Phenotype and Vice Versa

**Figure 2:**
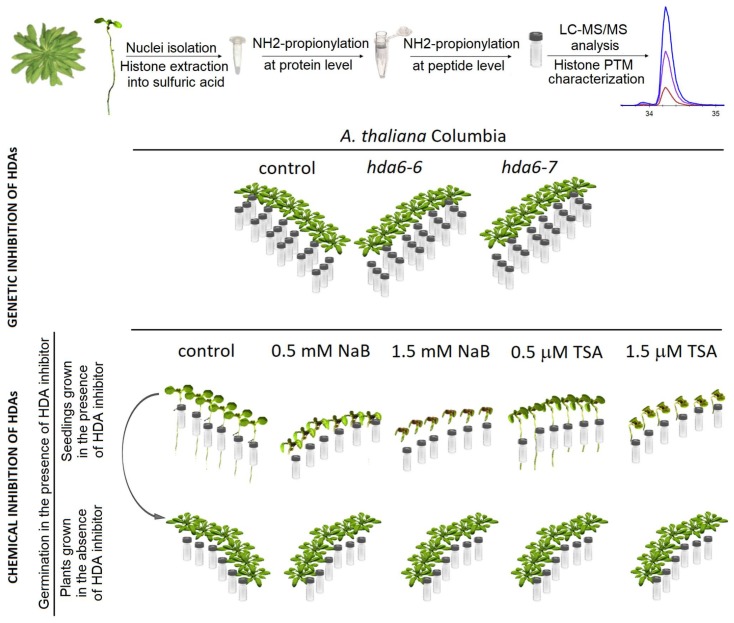
Experimental design. To quantify histone marks levels, histone proteins were extracted from seedlings germinated on different Petri dishes (6 replicates) or leaves of six independently cultivated plants. Samples were prepared for analysis by filter-aided sample preparation coupled with histone propionylation, digested with trypsin, and analyzed by LC-MS/MS. Leaves collected from 7-week-old *hda6* mutants and respective wild type plants were analyzed in a random order in three technical batches. Part of 7-day-old seedlings germinated in the presence of HDA inhibitors was harvested for proteomic analysis and part was transferred to the soil without HDA inhibitors and plants were grown for 7 weeks. These samples were analyzed in a random order in a single technical batch.

To examine changes in histone marks levels due to genetic or chemical inhibition of HDAs, the abundance of selected post-translationally modified histone peptides was evaluated from mass spectrometric analysis (experimental design is shown in Figure 2; see Materials and Methods for detailed description). As expected, peptide sequences corresponding to the N-termini of histones H3 and H4 exhibited high levels of PTMs. In particular, peptides of histone H3 included ten forms of K9STGGKAPR17 (H3K9–R17), three of K18QLATKAAR26 (H3K18–R26) and five of K27SAPATGGVKKPHR40 (H3K27–R40); peptides of histone H4 included eight forms of G4KGGKGLGKGGAKR17 (H4G4–R17). All these peptides possessed considerable levels of global acetylation, except for H3K27–R40 sequence. K27me2K36ac, with a relative abundance of <0.2%, represented the only acetylated form of H3K27–R40, whereas predominant forms of this peptide were dimethylated or monomethylated at K27 without any acetylation. Thus, the relative representation of acetylated forms of histone-peptides in HDA-deficient and control samples was evaluated for all above mentioned peptides except for H3K27–R40. The acetylation patterns of histone-peptides were statistically evaluated at three different levels: (1) comparison of overall acetylation status between HDA-deficient plants/seedlings and respective controls (i.e., the proportion of total abundance of all peptide forms containing acetyl group(s) relative to the total abundance of peptides without acetylation), (2) comparison of abundance of particular modified peptide forms between HDA-deficient plants/seedlings and respective controls (i.e., the proportional abundance of a particular peptide form containing acetyl group(s) relative to the abundance of all other peptides), and (3) comparison of the ratio of each pair of peptide forms in experimental and control groups.

**Figure 3:**
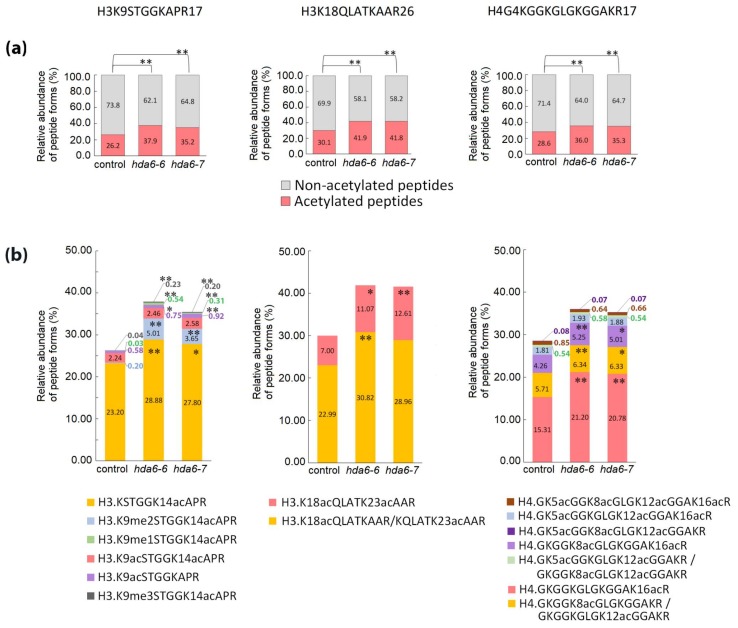
Relative abundance of peptide forms of histones H3 and H4 in leaves of wild type Arabidopsis plant (control) and *hda6* mutant lines (*hda6-6*, *hda6-7*). (**a**) The relative abundance of all peptide forms containing acetyl group(s) compared with that of peptides without acetylation. (**b**) The relative abundance of particular peptide forms containing acetyl group(s) compared with that of all other forms of respective peptide sequences. The levels of peptide forms were determined from the extracted ion chromatograms (XIC) peak areas. The significance of between-sample differences was assessed using t-tests, setting the significance threshold at ** *p* < 0.01 and * *p* < 0.05.

Compared to controls, significantly changed overall histone-peptide acetylation was observed in the leaves of both *hda6* lines (Figure 3a) corresponding to an increase by 9–11% in H3K9–R17 and H3K18–R26 acetylated peptide forms, and by 7–8% in H4G4–R17 acetylated peptide forms (Figure 3a and Table S1). Detailed investigation of modified peptide forms revealed significantly changed profiles in comparison to wild type controls, with a similar trend in both *hda6* lines.

Acetylation as the sole modification was detected as the prevailing form of H3K9–R17 modified peptides in control samples. In *hda6* lines, significantly increased levels of peptides with K14ac, including those containing me1 (methyl group), me2 or me3 at K9, were detected. On the other hand, the abundance of di-acetylated K9acK14ac peptide did not differ between *hda6* and control lines. Further, mono- and di-acetylated forms of H3K18–R26 peptide were elevated. In histone H4, mainly mono- and di-acetylated forms carrying acetylation in positions K8 and K16 were responsible for the hyperacetylation state in both *hda6-6* and *hda6-7* lines, while the abundance of peptides carrying three or four acetyl groups was comparable with control samples (Figure 3b). As expected, these changes were accompanied by significantly reduced levels of corresponding non-modified histone peptide sequences (Figure 3a; Tables S1 and S2). A disrupted balance of combinatorial patterns of histone modifications in *hda6* lines was also evident from the statistical evaluation of abundance of pairs of differentially modified histone peptide forms (Table S3). These results further indicate that HDA6 primarily affects acetylation at positions H3K14 and H4K16.

**Figure 4:**
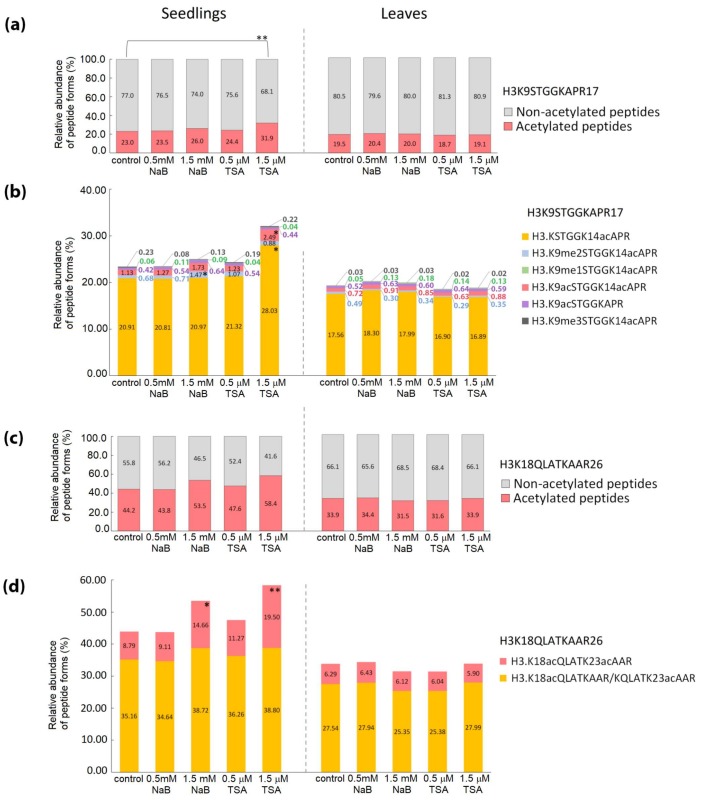
Relative abundance of peptide forms of histone H3 in seedlings germinated in the presence of HDA inhibitors, leaves of plants cultivated from these seedlings, and respective control samples. (**a**,**c**) The relative abundance of all peptide forms containing acetyl group(s) compared with that of peptides without acetylation. (**b**,**d**) The relative abundance of particular peptide forms containing acetyl group(s) compared with that of all other forms of respective peptide sequences. The levels of peptide forms were determined from the extracted ion chromatograms (XIC) peak areas. The significance of between-sample differences was assessed using t-tests, setting the significance threshold at ** *p* < 0.01 and * *p* < 0.05.

**Figure 5:**
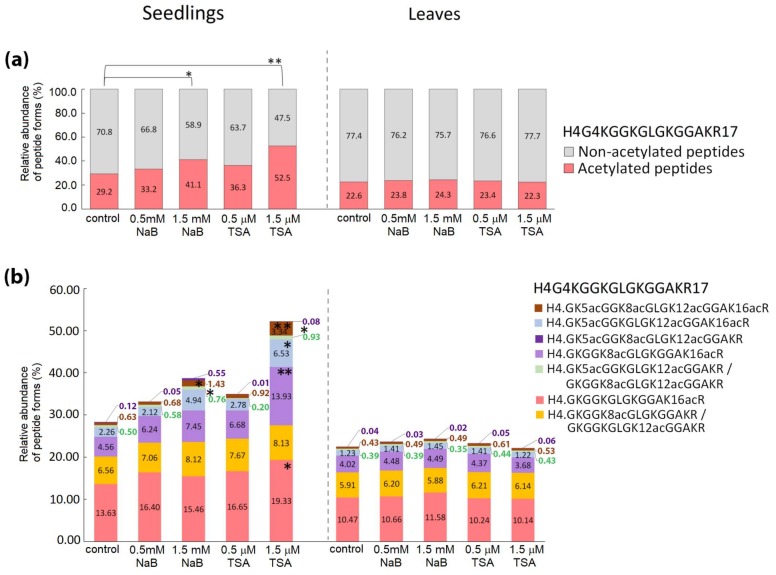
Relative abundance of peptide forms of histone H4 in seedlings germinated in the presence of HDA inhibitors, leaves of plants cultivated from these seedlings, and respective control samples. (**a**) The relative abundance of all peptide forms containing acetyl group(s) compared with that of peptides without acetylation. (**b**) The relative abundance of particular peptide forms containing acetyl group(s) compared with that of all other forms of respective peptide sequences. The levels of peptide forms were determined from the XIC peak areas. The significance of between-sample differences was assessed using t-tests, setting the significance threshold at ** *p* < 0.01 and * *p* < 0.05.

Increase in the levels of acetylated forms of histone-peptides in HDA inhibitor-treated seedlings was induced to a higher extent in histone H4 than in histone H3 (Figure 4 and Figure 5). Significant changes in histone mark levels were observed only in seedlings treated with higher doses of HDA inhibitors (Table S1). Treatment with 1.5 mM NaB and 1.5 μM TSA caused 12% and 23% increases in overall acetylations of H4G4–R17 peptide, respectively (Figure 5a). Both HDA inhibitors increased the number of forms carrying 2–4 acetyl groups. In the case of 1.5 μM TSA treatment, a higher abundance was also observed for K16-monoacetylated peptide (Figure 5b). Hyperacetylation of H4G4–R17 induced by both inhibitors was accompanied by a significant decrease in the levels of the non-modified form of this peptide (*p* < 0.05 and *p* < 0.001 for NaB and TSA, respectively). Regarding H3 histone, overall acetylation in H3K9-R17 peptide, corresponding to ~9% increase, was observed in seedlings exposed to 1.5 μM TSA (Figure 4a). Detailed evaluation of particular modified forms revealed significant changes in K14-mono- and di-acetylated H3K9–R17 peptides and di-acetylated H3K18–R26 peptide forms (Figure 4b,d). Interestingly, an increased level of the di-acetylated H3K18–R26 peptide, together with an increase in K9me2K14ac-containing peptide were detected in seedlings treated with 1.5 mM NaB (Figure 4b,d), although this was not reflected in the total levels of non-modified and acetylated forms of these peptides (Figure 4a,c). As supported by peptide pair statistics, NaB treatment caused moderate effects on histone epigenetic marks, as manifested by some increase in H3K9me2K14ac and di- and tetra-acetylated forms of H4G4-R17. On the contrary, TSA induced a comprehensive imbalance of solely acetylated epigenetic marks on histones H4 and H3 (Tables S2 and S3).

Seven weeks of growth in soil, thus in the absence of HDA inhibitors, allowed the plants to readjust all imbalanced modifications of histone peptides observed in seedlings to levels comparable to control plants (Figure 4 and Figure 5; Tables S1–S3).

## 3. Discussion

The growth of plants is modulated by regulatory pathways influencing vegetative development and response to environmental conditions. Members of the HDA family represent important epigenetic regulators involved in these comprehensive networks. Considering functional diversity of HDAs, which is determined by their subcellular localizations and different expression profiles, together with the fact that activity of these enzymes can be further affected by the interactions with other proteins, the response mechanisms appear to be highly complex [26].

A wide range of biochemical and molecular biological approaches have been contributed to deciphering the machinery of epigenetic regulations. The scope of knowledge in the field of histone epigenetic modifications is determined by the feasibility of respective analyses, which are challenging due to the complex pattern of histone PTMs and the presence of histone variants.

In agreement with previously published data [11,12,25], mutations in the *HDA6* gene did not affect the phenotype of plants during early stages of growth. On the other hand, delayed flowering and the onset of leaf senescence in *hda6-6* and *HDA6-RNAi* plants have been reported, indicating that HDA6 affected rather later periods of plant development [25]. In these plants, except for an increase in the global level of H3 acetylation, H3 hyperacetylation was detected in the *FLC* gene locus, with *FLC* encoding a transcription factor that controls the transition from vegetative to reproductive developmental stages. Based on these data, we focused on analysis of 7-week-old *hda6* plants. Our MS-based approach allowed us to distinguish and quantify a number of post-translationally modified histone peptide forms. Although *hda6* plants exhibited no obvious morphological changes, significant alterations were found at the level of histone modifications (Figure 3). Based on circumstantial analyses, Earley et al. deduced H3K14, H4K5 and H4K12 as target sites for HDA6 deacetylase activity [13]. We confirmed the position K14 of histone H3 as a dominant HDA6 target, and demonstrated that K14 deacetylation is not dependent on PTMs present at K9. In addition, the lack of HDA6 also led to hyperacetylation at positions H3K18 and H3K23 (Figure 3b). Instead of H4K5 and H4K12, as reported by Earley and coworkers [13], acetylation at H4K8 and primarily at H4K16 were found to be affected in *hda6* lines. Importantly, levels of tri- and tetra-acetylated forms of histone H4 peptides were not elevated, indicating that plant cells tend to minimize hyperacetylation due to an *HDA6* gene mutation using redundant activities of other HDAs to ensure plant survival and prevent growth and developmental defects.

As a parallel approach for modulating the activity of HDAs, we germinated Arabidopsis seeds in the presence of TSA or NaB. According to Ueda’s classification [27], NaB is a selective inhibitor of class I HDAs (HDA6, HDA7, HDA9, HDA19) and class II (HDA5, HDA14, HDA15, HDA18) while TSA, as a pan-inhibitor, inhibits HDAs of classes I and II and also class IV (HDA2) and some unclassified HDAs (HDA8, HDA10, HDA17). Higher variability of histone PTMs in the biological replicates of TSA-treated *A. thaliana* leaves was previously observed in a proteomic study, and accordingly, a relatively low number of specific sites with modulated acetylation state were identified [28]. Analysis of six biological replicates (seedlings cultivated on six Petri dishes) revealed significantly changed levels of histone PTMs in samples treated with higher doses of inhibitors (Figure 4 and Figure 5). Apparently, variability in the data of NaB- and TSA-exposed samples was higher compared to results of analyses of *hda6* mutants (Tables S1–S3), which obviously reflected the heterogeneous phenotype of seedlings germinated in the presence of HDA inhibitors (Figure 1a). In this context, it is necessary to stress the strong phenotypic defects of 7-day-old seedlings germinated in the presence of HDA inhibitors (Figure 1a,b).

However, and rather surprisingly, the extent of changes in histone acetylation did not correlate with morphological changes (Figure 4 and Figure 5). Specifically, NaB-exposed seedlings had severely disturbed germination but changes in histone acetylation were remarkably lower than in seedlings treated with TSA, exhibiting a milder phenotype. The modest increase in histone acetylation levels in NaB-treated seedlings correlated with the lower number of HDAs that were inhibited by this drug, compared to TSA. The strong effect of NaB on the processes of early plant development is thus probably related to the modes of action of this drug other than HDA inhibition. The phenotype comparable with wild type plants, together with highly impaired histone acetylation status in *hda6* mutants, i.e., plants with loss of function of a single HDA, further supports this scenario. Indeed, the effect of HDA inhibitors on protein partners that are independent of HDA activity has previously been reported in several studies in mammalian cells [29,30,31,32]. Another possibility is that due to reduced HDA activity caused by HDA inhibitors, metabolic pathways that involve acetylated target proteins other than histones might be influenced. Acetylated lysines were found in a number of proteins localized in diverse subcellular compartments in both plants and mammals. Potential non-histone targets of RPD3-like enzymes in Arabidopsis were investigated in a recently published comprehensive proteomic study [28].

Detailed analysis revealed increased length and density of root hairs as striking features of TSA-treated seedlings (Figure 1c). Rapidly and reversibly altered patterns of root hair development after TSA treatment were previously reported and associated with histone hyperacetylation-related expression of *CPC*, *GL2*, and *WER* developmental patterning genes [33]. According to this study, HDA18 is a key component involved in the regulation of root hair formation. As activity of this class II HDA is supposed to be influenced by both TSA and NaB inhibitors, a broader inhibitory capacity of TSA may somehow augment the final effect. On the other hand, the process of germination was retarded by NaB to the extent of making the proper phenotypic analysis of roots impossible. Importantly, the effect of HDA inhibitors on the phenotype as well as on histone acetylation status was transient, since subsequent cultivation of plants in the soil conferred features comparable with control plants (Figure 1d, Figure 4 and Figure 5).

The regulatory role of HDAs in crucial developmental processes has been convincingly demonstrated using various plant species (reviewed in [34]). Here, we performed comprehensive proteomic characterization of histone PTMs to obtain new information on the relationship between phenotypic features and the pattern of histone modifications in *A. thaliana* plants with abolished HDA activities. Our data demonstrate the complexity not only of HDA action but also of effects of HDA inhibitors, as the ability of these drugs to influence plant growth and development does not seem to be limited to modulation of histone acetylation.

## 4. Materials and Methods

### 4.1. Cultivation of Plants, Monitoring of Phenotype

*Arabidopsis thaliana* ecotype Col-0 (Nottingham Arabidopsis Stock Centre, Nottingham, UK) seeds collected from a set of parent plants were ethanol sterilized and placed on Petri dishes containing half-strength Murashige-Skoog medium (Duchefa Biochemicals, Haarlem, The netherlands) supplemented with 0.8% plant agar. The medium was then supplemented with inhibitors of HDAs, 0.5 mM NaB (Sigma-Aldrich, St Lous, MO, USA), 1.5 mM NaB, 0.5 μM TSA (Sigma-Aldrich), and 1.5 μM TSA. Seeds were vernalized for 3 days at 4 °C and germinated for 7 days in phytotrons under short day conditions (8 h light, 21 °C, illumination 100 μmol m^−2^ s^−1^; 16 h dark, 19 °C). The lengths of roots of at least 30 seedlings cultivated on control medium or exposed to HDA inhibitors were measured using ImageJ software (Fiji; [35]), and results were statistically evaluated by the Mann–Whitney U test. A proportion of the seedlings was then harvested and a proportion was transferred to the soil. Plants were grown for seven weeks in the phytotrons under short day conditions; growth progress was monitored weekly.

Seeds of the *hda6-7* mutant line were germinated and plants were cultivated as described above. Plants were genotyped using a primer pair distinguishing a 37-bp deletion in the mutant as described in [12]. Seeds of *hda6-6* were germinated on half-strength Murashige-Skoog medium supplemented with 25 mg·mL^−1^ hygromycin, plants were then cultivated in soil as described above. Seeds of both *hda6* mutants were kindly provided by Frederic Pontvianne (CNRS and Universite’ de Perpignan Via Domitia, Perpignan, France).

For LC-MS/MS analysis, histones extracted from seedlings germinated on six Petri dishes and leaves of six independently cultivated plants were taken. The samples isolated from leaves of 7-week old *hda6* mutant lines and wild type Col-0 plants were measured in random order in three technical batches. Seven-day-old seedlings germinated in the presence of HDA inhibitors and 7-week-old plants grown in the soil from these seedlings, as well as corresponding control samples, were analyzed in random order in a single technical batch. Experimental design is schematically illustrated in Figure 2.

### 4.2. Histone Isolation and Preparation of Samples for Mass Spectrometry

The procedures used for extraction and chemical derivatization of plant histones have been previously described [24]. Nuclei isolated from ~500 mg of plant tissues were resuspended in the nuclei lysis buffer (50 mM Tris-HCl pH 8.0, 100 mM NaCl, 3 mM EDTA, 1% CHAPS, 0.1 μM PMSF, 45 mM NaB, and 10 μL/mL of P9599 protease inhibitor cocktail from Sigma-Aldrich), incubated for 1 h on ice, and centrifuged (8 min, 10,000× *g*, 4 °C). The pellets were resuspended in 200–400 µL of ice-cold 0.2 M H_2_SO_4_ and incubated overnight with shaking at 4 °C. Samples were centrifuged (8 min, 16,100× *g*, 4 °C) and supernatants containing histone proteins were collected. Sixteen μg of plant histone extract in sulfuric acid was subjected to a double round of propionic anhydride derivatization. After pH adjustment to 8–9 with NH_4_OH, 10 μL of propionylation reagent (1:3 mixture of propionic anhydride and acetonitrile (MeCN); both from Sigma-Aldrich) were added to the samples and incubated in a thermomixer (37 °C, 700 rpm, 20 min). The sample volumes were reduced in a Savant SPD121P concentrator (SpeedVac; Thermo Fisher Scientific, Waltham, MA, USA) to 5 μL. For the second round of propionylation, samples were diluted with 50% (*v/v*) MeCN to 20 μL and propionylation was carried out using the same protocol. Samples were diluted with 300 μL of 8 M urea (pH 8.5), placed in a YM-10 Microcon filter unit (Merck Millipore, Burlington, MA, USA), centrifuged (45 min, 14,000× *g*, 25 °C), washed two times with 200 μL of 8 M urea and three times with 100 μL of 100 mM ammonium bicarbonate (ABC; 45 min, 14,000× *g*, 25 °C). Trypsin (Promega, Madison, WI, USA) diluted in 50 μL of 100 mM ABC was added in a 1:40 (enzyme:protein) ratio. Digestion was carried out overnight at 37 °C. The digest was collected by centrifugation (10 min, 14,000× *g*, 25 °C), subjected to two additional washes with 50 μL of 100 mM ABC and concentrated using the SpeedVac to a volume of ~20 μL. One μL of NH_4_OH and 5 μL of the propionylation reagent prepared by mixing propionic anhydride with MeCN in a 1:3 ratio were added. The pH was adjusted to 8–9 with NH_4_OH, samples were incubated in thermomixer at 37 °C at 700 rpm for 20 min, then the volume was reduced in the SpeedVac to 5 μL. For the second round of propionylation, samples were diluted with 50% (*v/v*) MeCN to a volume of 20 μL and the process was carried out using the same protocol. The samples were diluted with 0.1% formic acid to a volume of 100 μL. Labeled histones were desalted using a Hypersep SpinTip C-18 column (Thermo Fisher Scientific).

### 4.3. Mass Spectrometric Analysis, Database Searches and Quantification of Histone Peptide Forms

Mass spectrometric analysis of histone peptides and following data processing was done as described previously [24]. Propionylated peptides were measured using LC-MS/MS. The LC-MS/MS equipment consisted of an RSLCnano system, equipped with an X-Bridge BEH 130 C18 trap column (3.5 μm particles, 100 μm × 30 mm; Waters), and an Acclaim Pepmap100 C18 analytical column (3 µm particles, 75 μm × 500 mm; Thermo Fisher Scientific), coupled to an Orbitrap Elite hybrid spectrometer (Thermo Fisher Scientific) equipped with a Digital PicoView 550 ion source (New Objective, Woburn, MA, USA) using a PicoTip SilicaTip emitter (FS360-20-15-N-20-C12), and an Active Background Ion Reduction Device. Prior to LC separation, tryptic digests were online concentrated on a trap column. The mobile phase consisted of 0.1% formic acid in water (A) and 0.1% formic acid in 80% acetonitrile (B), with the following proportions of B: 1% for 3 min at 500 nL/min, then with a switch to 300 nL/min for the next 2 min, increasing linearly from 1% to 70% over 85 min, 70–85% over 20 min and followed by isocratic washing at 85% B for 10 min. Equilibration of the trapping and separation columns was carried out using 99:1 (mobile phase A:B; flow rate 500 nL/min) prior to sample injection to the sample loop. The analytical column outlet was directly connected to the ion source of the MS. MS data were acquired using a data-dependent strategy selecting up to the top 10 precursors based on precursor abundance in a survey scan (350–2000 m/z). The resolution of the survey scan was 60,000 (400 m/z) with a target value of 1 × 10^6^, one microscan and maximum injection time of 1000 ms. HCD MS/MS spectra were acquired with a target value of 50,000 and resolution of 15,000 (400 m/z). The maximum injection time for MS/MS was 500 ms. Dynamic exclusion was enabled for 45 s after one MS/MS spectrum acquisition and early expiration was disabled. The isolation window for MS/MS fragmentation was set to 2 m/z.

The RAW mass spectrometric data files were analyzed using Proteome Discoverer software (Thermo Fisher Scientific; version 1.4) with an in-house Mascot search engine (Matrixscience, London, UK; version 2.6) to compare acquired spectra with entries in the UniProtKB Arabidopsis thaliana protein database (version 2017_11; 27567 protein sequences; downloaded from ftp://ftp.uniprot.org/pub/databases/uniprot/current_release/knowledgebase/reference_proteomes/Eukaryota/UP000006548_3702.fasta.gz), cRAP contaminant database (downloaded from http://www.thegpm.org/crap/) and in-house histone database (version 2017_02; 71 protein sequences). Mass tolerances for peptides and MS/MS fragments were 7 ppm and 0.025 Da, respectively. Semi-Arg-C for enzyme specificity allowing up to two missed cleavages was set. For searches against cRAP and UniProtKB Arabidopsis thaliana databases, the variable modification settings were oxidation (M), deamidation (N, Q), acetylation (K, protein N-term) and propionylation (K, N-term), while for histone database searches they were acetylation (K, protein N-term), methylation (K, R), dimethylation (K), trimethylation (K), phosphorylation (S, T), and propionylation (K, N-term, S, T, Y). The abundance of histone peptides was quantified automatically using Proteome Discoverer 1.4 software. Only peptides with statistically significant peptide scores (*p* < 0.01) were considered. The peak area corresponding to each precursor ion was calculated from the extracted ion chromatograms (XICs) using the Precursor Ions Area Detector node. Selected histone peptide identifications were manually verified and quantified from the peak areas derived from the XICs using Skyline 3.6 software, including identification alignment across the raw files based on retention time and m/z.

The mass spectrometry proteomics data were deposited to the ProteomeXchange Consortium via the PRIDE [36] partner repository with the dataset identifier PXD014739.

### 4.4. Data Analysis

The peak areas corresponding to post-translationally modified forms of individual histone peptide were treated as compositions and Aitchison’s methodology [37] based on log-ratios was applied in the statistical evaluation. First, the missing values were imputed by iterative least trimmed squares regression [38] and areas were transformed to relative abundances (percentages). Then, acetylated and non-acetylated forms of each peptide were amalgamated. All forms of acetylated/non-acetylated histones were processed as follows. The technical replicates were aggregated by geometric mean and closure. In order to fully exploit the statistical significance of differences in relative abundances of individual forms of peptides, multiple transformations, defined for compositional data, were applied. Note that in compositional data, the relative abundances of individual parts were not directly comparable due to the constant sum constraint leading to a spurious negative correlation. The log2 ratio of amalgamated acetylated and non-acetylated forms was calculated (alr-transformation of 2-parts composition) and the t-test was used to compare means of the ratios between corresponding pairs of experimental and control groups. The relative abundances of all individual peptide forms were first ilr-transformed and compared by Hotelling’s T2 test to globally assess the differences in the distribution of forms of each peptide. Later, for each peptide, the log2 ratio of relative abundance of one form to the sum of relative abundances of all other forms was calculated and the *t*-test was applied to assess the difference in each individual form. Finally, log2 ratios of all pairs of peptide forms were calculated and compared by *t*-test. The data analysis was performed in R version 3.5.0 [39].

## Supplementary Materials

Supplementary materials can be found at https://www.mdpi.com/1422-0067/20/20/5093/s1.

## Abbreviations

HAT	Histone acetyltransferase
HDA	Histone deacetylase
NaB	Sodium butyrate
MS	Mass spectrometry
PTM	Post-translational modification
TSA	Trichostatin A

## References

[1] Brownell J.E., Allis C.D. (1996). Special HATs for special occasions: Linking histone acetylation to chromatin assembly and gene activation. Curr. Opin. Genet. Dev..

[2] Kuo M.H., Allis C.D. (1998). Roles of histone acetyltransferases and deacetylases in gene regulation. Bioessays.

[3] Chen Z.J., Pikaard C.S. (1997). Epigenetic silencing of RNA polymerase I transcription: A role for DNA methylation and histone modification in nucleolar dominance. Genes Dev..

[4] Kadosh D., Struhl K. (1998). Targeted recruitment of the Sin3-Rpd3 histone deacetylase complex generates a highly localized domain of repressed chromatin in vivo. Mol. Cell Biol..

[5] Ning Y.Q., Chen Q., Lin R.N., Li Y.Q., Li L., Chen S., He X.J. (2019). The HDA19 histone deacetylase complex is involved in the regulation of flowering time in a photoperiod-dependent manner. Plant J..

[6] Tanaka M., Kikuchi A., Kamada H. (2008). The Arabidopsis histone deacetylases HDA6 and HDA19 contribute to the repression of embryonic properties after germination. Plant Physiol..

[7] Tian L., Chen Z.J. (2001). Blocking histone deacetylation in Arabidopsis induces pleiotropic effects on plant gene regulation and development. Proc. Natl. Acad. Sci. USA.

[8] Zheng M., Liu X.B., Lin J.C., Liu X.Y., Wang Z.Y., Xin M.M., Yao Y.Y., Peng H.R., Zhou D.X., Ni Z.F. (2019). Histone acetyltransferase GCN5 contributes to cell wall integrity and salt stress tolerance by altering the expression of cellulose synthesis genes. Plant J..

[9] Pandey R., Muller A., Napoli C.A., Selinger D.A., Pikaard C.S., Richards E.J., Bender J., Mount D.W., Jorgensen R.A. (2002). Analysis of histone acetyltransferase and histone deacetylase families of Arabidopsis thaliana suggests functional diversification of chromatin modification among multicellular eukaryotes. Nucleic Acids Res..

[10] Yang X.J., Seto E. (2003). Collaborative spirit of histone deacetylases in regulating chromatin structure and gene expression. Curr. Opin. Genet. Dev..

[11] Murfett J., Wang X.J., Hagen G., Guilfoyle T.J. (2001). Identification of arabidopsis histone deacetylase HDA6 mutants that affect transgene expression. Plant Cell.

[12] Aufsatz W., Mette M.F., van der Winden J., Matzke M., Matzke A.J. (2002). HDA6, a putative histone deacetylase needed to enhance DNA methylation induced by double-stranded RNA. EMBO J..

[13] Earley K., Lawrence R.J., Pontes O., Reuther R., Enciso A.J., Silva M., Neves N., Gross M., Viegas W., Pikaard C.S. (2006). Erasure of histone acetylation by Arabidopsis HDA6 mediates large-scale gene silencing in nucleolar dominance. Genes Dev..

[14] Liu X.C., Yu C.W., Duan J., Luo M., Wang K.C., Tian G., Cui Y.H., Wu K.Q. (2012). HDA6 Directly Interacts with DNA Methyltransferase MET1 and Maintains Transposable Element Silencing in Arabidopsis. Plant Physiol..

[15] Probst A.V., Fagard M., Proux F., Mourrain P., Boutet S., Earley K., Lawrence R.J., Pikaard C.S., Murfett J., Furner I. (2004). Arabidopsis histone deacetylase HDA6 is required for maintenance of transcriptional gene silencing and determines nuclear organization of rDNA repeats. Plant Cell.

[16] Chen L.T., Luo M., Wang Y.Y., Wu K.Q. (2010). Involvement of Arabidopsis histone deacetylase HDA6 in ABA and salt stress response. J. Exp. Bot..

[17] Wang Y.Z., Hu Q., Wu Z.J., Wang H., Han S.M., Jin Y., Zhou J., Zhang Z.F., Jiang J.F., Shen Y. (2017). HISTONE DEACETYLASE 6 represses pathogen defence responses in Arabidopsis thaliana. Plant Cell Environ..

[18] Yu C.W., Liu X.C., Luo M., Chen C.Y., Lin X.D., Tian G., Lu Q., Cui Y.H., Wu K.Q. (2011). HISTONE DEACETYLASE6 Interacts with FLOWERING LOCUS D and Regulates Flowering in Arabidopsis. Plant Physiol..

[19] Finnin M.S., Donigian J.R., Cohen A., Richon V.M., Rifkind R.A., Marks P.A., Breslow R., Pavletich N.P. (1999). Structures of a histone deacetylase homologue bound to the TSA and SAHA inhibitors. Nature.

[20] Cousens L.S., Gallwitz D., Alberts B.M. (1979). Different Accessibilities in Chromatin to Histone Acetylase. J. Biol. Chem..

[21] Ma X.J., Zhang C., Zhang B., Yang C.P., Li S.J. (2016). Identification of genes regulated by histone acetylation during root development in Populus trichocarpa. BMC Genom..

[22] Earley K.W., Shook M.S., Brower-Toland B., Hicks L., Pikaard C.S. (2007). In vitro specificities of Arabidopsis co-activator histone acetyltransferases: Implications for histone hyperacetylation in gene activation. Plant J..

[23] Pagano A., Araujo S.D., Macovei A., Dondi D., Lazzaroni S., Balestrazzi A. (2019). Metabolic and gene expression hallmarks of seed germination uncovered by sodium butyrate in Medicago truncatula. Plant Cell Environ..

[24] Ledvinová D., Mikulášek K., Kuchaříková H., Brabencová S., Fojtová M., Zdráhal Z., Lochmanová G. (2018). Filter-Aided Sample Preparation Procedure for Mass Spectrometric Analysis of Plant Histones. Front. Plant Sci..

[25] Wu K., Zhang L., Zhou C., Yu C.W., Chaikam V. (2008). HDA6 is required for jasmonate response, senescence and flowering in Arabidopsis. J. Exp. Bot..

[26] Ma X., Lv S., Zhang C., Yang C. (2013). Histone deacetylases and their functions in plants. Plant Cell Rep..

[27] Ueda M., Matsui A., Tanaka M., Nakamura T., Abe T., Sako K., Sasaki T., Kim J.M., Ito A., Nishino N. (2017). The Distinct Roles of Class I and II RPD3-Like Histone Deacetylases in Salinity Stress Response. Plant Physiol..

[28] Hartl M., Füßl M., Boersema P.J., Jost J.O., Kramer K., Bakirbas A., Sindlinger J., Plöchinger M., Leister D., Uhrig G. (2017). Lysine acetylome profiling uncovers novel histone deacetylase substrate proteins in *Arabidopsis*. Mol. Syst. Biol..

[29] Činčárová L., Lochmanová G., Nováková K., Šultesová P., Konečná H., Fajkusová L., Fajkus J., Zdráhal Z. (2012). A combined approach for the study of histone deacetylase inhibitors. Mol. Biosyst..

[30] Chang J., Varghese D.S., Gillam M.C., Peyton M., Modi B., Schiltz R.L., Girard L., Martinez E.D. (2012). Differential response of cancer cells to HDAC inhibitors trichostatin A and depsipeptide. Br. J. Cancer.

[31] Krämer O.H., Zhu P., Ostendorff H.P., Golebiewski M., Tiefenbach J., Peters M.A., Brill B., Groner B., Bach I., Heinzel T. (2003). The histone deacetylase inhibitor valproic acid selectively induces proteasomal degradation of HDAC2. EMBO J..

[32] Marks P.A., Xu W.S. (2009). Histone deacetylase inhibitors: Potential in cancer therapy. J. Cell Biochem..

[33] Xu C.R., Liu C., Wang Y.L., Li L.C., Chen W.Q., Xu Z.H., Bai S.N. (2005). Histone acetylation affects expression of cellular patterning genes in the Arabidopsis root epidermis. Proc. Natl. Acad. Sci. USA.

[34] Chen D.H., Huang Y., Jiang C., Si J.P. (2018). Chromatin-Based Regulation of Plant Root Development. Front. Plant Sci..

[35] Schindelin J., Arganda-Carreras I., Frise E., Kaynig V., Longair M., Pietzsch T., Preibisch S., Rueden C., Saalfeld S., Schmid B. (2012). Fiji: An open-source platform for biological-image analysis. Nat. Methods.

[36] Vizcaíno J.A., Csordas A., Del-Toro N., Dianes J.A., Griss J., Lavidas I., Mayer G., Perez-Riverol Y., Reisinger F., Ternent T. (2016). 2016 update of the PRIDE database and its related tools. Nucleic Acids Res..

[37] Aitchison J. (1982). The Statistical-Analysis of Compositional Data. J. Roy. Stat. Soc. B Met..

[38] Hron K., Templ M., Filzmoser P. (2010). Imputation of missing values for compositional data using classical and robust methods. Comput. Stat. Data Anal..

[39] R Core Team R: A Language and Environment for Statistical Computing, R Foundation for Statistical Computing: 2018. http://www.R-project.org.

